# Effect of posture on pulmonary function and oxygenation after fast-tracking video-assisted thoracoscopic surgery (VATS) lobectomy: a prospective pilot study

**DOI:** 10.1186/s13741-021-00199-z

**Published:** 2021-09-02

**Authors:** Lin Huang, Henrik Kehlet, René Horsleben Petersen

**Affiliations:** 1 Department of Cardiothoracic Surgery, Copenhagen University Hospital, Rigshospitalet, Copenhagen, Denmark; 2 Section of Surgical Pathophysiology, Copenhagen University, Rigshospitalet, Blegdamsvej 9, DK-2100 Copenhagen, Denmark

**Keywords:** Pulmonary function, Oxygenation, Enhanced recovery, Video-assisted thoracoscopic surgery, Mobilisation

## Abstract

**Background:**

Minimally invasive surgery combined with enhanced recovery programmes has improved outcomes after lung cancer surgery and where early mobilisation may be an important factor. However, little is known about pulmonary function and oxygenation during mobilisation after video-assisted pulmonary lobectomy. The aim of this prospective pilot cohort study was to explore the effect of postural changes (from supine to sitting to standing) on pulmonary function and oxygen saturation in a well-defined enhanced recovery programmes setting after video-assisted thoracoscopic surgery lobectomy.

**Methods:**

A total of 24 patients were evaluated daily for postoperative pain score, pulmonary function (forced expiratory volume 1 s) and oxygen saturation in supine, sitting and standing position from 6 h after surgery to 6 h after chest drain removal.

**Results:**

Mobilisation from supine to standing position showed a significant 7.9% increase (*p* = 0.04) in forced expiratory volume in 1 s percentage and oxygen saturation about 1.8% (*p*< 0.001) without increasing pain (*p* = 0.809).

**Conclusions:**

Early mobilisation should be encouraged to enhance recovery after video-assisted thoracoscopic surgery lobectomy by increasing lung function and oxygen delivery.

**Trial registration:**

• Name of the registry: clinicaltrials.gov

• Trial registration number: NCT04508270

• Date of registration: August 11, 2020

## Introduction

Pulmonary function is known to decrease after major surgery including thoracic surgery (Craig 1981) with potential consequences on risk of pulmonary and other complications. Although postoperative changes in pulmonary function may be related to pain and the surgical stress response (inflammation), body position may also be important, since moving from supine to sitting or standing position may improve pulmonary function (Craig et al. 1971, Meyers et al. 1975, Hsu and Hickey 1976, Bonnet et al. 1988).

Although the concept of “fast-track” or “enhanced recovery” surgery (ERAS) from the beginning included early mobilisation (Kehlet 1997), little information is available on the effect of postural changes on pulmonary function and oxygenation in ERAS programmes including minimal invasive surgery (Balvardi et al. 2021), despite initial observations in non-ERAS open abdominal surgery showing improved oxygen saturation when moving from supine to standing position (Mynster et al. 1996).

Improvements in care and surgical technique with minimal invasive thoracoscopic surgery (VATS) have improved pulmonary outcomes combined with ERAS implementation (Batchelor et al. 2019), but specific studies on the role of posture on lung function and oxygenation are not available.

Consequently, the aim of this study was to explore the effect of postural changes on pulmonary function and oxygen saturation in a well-defined ERAS setting after VATS lobectomy.

## Methods

### Study design and patient selection

The study was exploratory, prospective and observational, adhering to the Strengthening the Reporting of Observational Studies (STROBE) (Gharaibeh et al. 2014) and approved by Danish Regional Ethics Committee (H-20041481) and registered in the Danish Data Protection Agency (P-2020-791) and ClinicalTrials.gov (NCT04508270). Written consent was obtained from all participants.

Patients (age ≥ 18 years) who spoke Danish and were scheduled for VATS lobectomy from September 08, 2020, to December 17, 2020, at the department of Cardiothoracic Surgery, Copenhagen University Hospital, Rigshospitalet, were approached for inclusion. Exclusion criteria included bilobectomy, segmentectomy, wedge resection, lobectomy combined with other surgical procedures, thoracotomy, unable to stand up, unable to discontinue oxygen therapy in the first postoperative 6 h or unwilling to complete lung function or oxygen saturation test. All patients received a standard perioperative care with intubation with intravenous inhalation anaesthesia and multimodal pain management as published previously (Hansen and Petersen 2012, Wildgaard et al. 2012). Since no similar study has been published, we did not conduct a formal power calculation. Given reasonability and feasibility (Hertzog 2008), we planned to include 24 patients, viewed as a detailed pilot study before embarking on a large outcome trial.

### Collection of demographics and clinical data

Age, sex, body mass index (BMI), American Society of Anaesthesiologists classification (ASA), comorbidity [Charlson Comorbidity Index (CCI)], smoking situation (never smoke, current smoker or former smoker), duration of surgery, blood loss, duration of chest drainage and length of hospital stay (LOS) were extracted from the electronic medical records (Epic, Madison, Wisconsin).

### Measurement of pulmonary function, oxygen saturation and postoperative pain

When the patient was awake without continuous oxygen therapy after 6 h from the end of surgery (PO6h), pulmonary function and SpO_2_ was measured in supine, sitting and standing position. Simultaneously, postoperative pain was evaluated.

The process was repeated on the morning of the postoperative day 1 (POD 1), POD 2 and 6 h after chest drain removal (PODR6). After 15 min rest, the measurements were done in supine position, followed by a 5 min interval before changing to the next posture. Each patient was assessed three times with every posture, using the best value for calculation.

SpO_2_ was monitored via an oximeter, Vitalograph® copd-6™ (Model 4000 respiratory monitor, Vitalograph, Ennis, Ireland) probing left index finger. A respirometer, PureSAT® (Model 2500 pulse oximeter, Nonin medical, Inc., Plymouth, MN, USA), was used to assess pulmonary function, including forced expiratory volume in 1 s (FEV_1_ and FEV_1_%). Postoperative pain was measured by numeric rating scale (NRS) with eleven-point numeric range (from ‘0’ no pain to ‘10’ worst pain).

All data was anonymously stored on Research Electronic Data Capture (REDCap™) tool (Harris et al. 2009).

### Statistical analysis

The distribution of continuous variables was evaluated via Kolmogorov-Smirnov and Shapiro-Wilk test. Variables with normal or non-normal distribution were presented as mean and standard deviation (SD) or median and interquartile range (IQR), respectively. Categorical variables were showed using frequencies (percentage). A mixed-model analysis of variance (ANOVA) with Tukey correction was used to assess differences of repeated measurement in SpO_2_, FEV_1_% and NRS (supine, sitting and standing at PO6h, POD 1, POD 2 and PODR6. A *p* value of < 0.05 was chosen as statistically significant. The statistical software SPSS (version 25.0, IBM-SPSS Inc., Armonk, NY) and R (version 4.0.3, R Foundation for Statistical Computing, Vienna, Austria) was used for analyses.

## Results

**Fig. 1:**
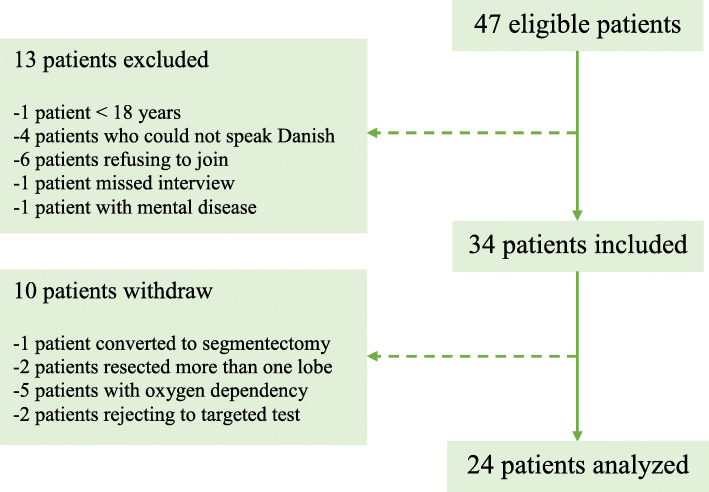
The flowchart of patients enrolled, included and analysed

Of 47 eligible patients, 24 patients meet the inclusion criteria for final analysis (Fig. 1).

Patient demographics and clinical characteristics are shown in Table 1 and are not deviating from a conventional series of VATS lobectomy (Hansen and Petersen 2012). Patient median age (IQR [range]) was 71 (66, 72 [57, 81]) years. Mean (SD) BMI was 26.9 (5.4) kg/m^2^. Most patients had a smoking history, including 15 (62.5%) former smokers and 4 (16.7%) current smokers. The median (IQR [range]) of CCI was 1.5 (1.0, 3.0 [0, 9.0]). Mean (SD) duration of surgery was 96 (22) min and blood loss 47 (74) ml. Of note, duration of chest drainage was short (median 1.0 days, mean 1.5 days) as well as length of hospitalisation (LOS) (median 2.0 days, mean 2.2 days).

**Table 1:** Participants demographic and clinical characteristics

Variable	
Age^a^, years	71 (66, 72 [57, 81])
Sex^c^	
Male	12 (50)
Female	12 (50)
BMI^b^, kg/m^2^	26.9 (5.4)
ASA^c^	
1	0
2	3 (12.5)
3	21 (87.5)
> 3	0
Smoke status^c^	
No smoke	5 (20.8)
Former smoker	15 (62.5)
Current smoker	4 (16.7)
FEV_1_%^b^	92.5 (19.1)
SpO_2_^b^, %	97.1 (1.6)
Chronic pain^c^	2 (8.3)
Charlson Comorbidity index^a^	1.5 (1.0, 3.0 [0, 9.0])
Duration of surgery^b^, mins	96 (22)
Blood loss^b^, ml	47 (74)
Duration of chest drainage^a^, days	1.0 (1.0, 2.0 [0, 7.0])
Length of hospital stay^a^, days	2.0 (1.0, 2.8 [1.0, 8.0])

*ASA* American Society of Anaesthesiologists classification, *BMI* body mass index, *FEV*_*1*_*%* percentage of predicted forced expiratory volume in 1 s value, *SpO*_*2*_ oxygen saturation

^a^Value presents as median (interquartile range [range])

^b^Value presents as mean (standard deviation)

^c^Value presents as frequency (percentage)

**Fig. 2:**
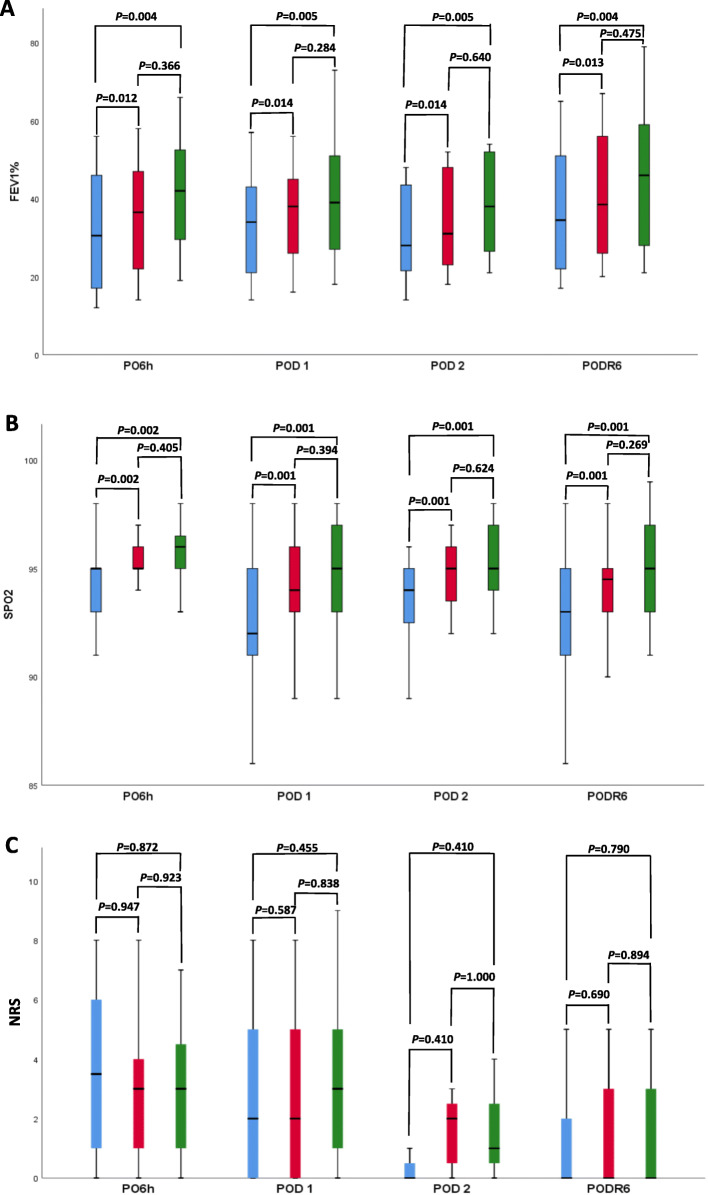
Postoperative changes in **A** percentage of predicted forced expiratory volume in 1 s value (FEV_1_%), **B** oxygen saturation (SpO_2_), **C** numerical rating scale (NRS) for assessing postoperative pain under three positions-supine (blue box), sitting (red box) and standing (green box)-within after 6 h from the end of surgery (PO6h), postoperative day 1 (POD 1), POD 2 and 6 h after chest drain removal (PODR6). Data are median with a box from first quartile to third quartile and a vertical line showing range

Postoperative changes in FEV_1_%, SpO_2_ and postoperative pain (NRS) are detailed in Table 2 and Fig. 2. The data on changes in FEV_1_%, SpO_2_ and NRS are shown in Fig. 2. The overall results showed a significant increase in all parameters after mobilisation from supine to standing, except pain (mean FEV_1_% 7.9%, 95% CI 2.08 to 12.96, *P* = 0.04; mean SpO_2_ 1.8%, 95% CI 0.99 to 2.70, *P* < 0.001; mean NRS 0.3, 95% CI − 0.62 to 1.06, *P* = 0.809).

**Table 2:** Postoperative lung function, oxygen saturation and pain score

Variables	Overall			PO6h			POD1			POD2			PODR6		
	Supine	Sitting	Standing	Supine	Sitting	Standing	Supine	Sitting	Standing	Supine	Sitting	Standing	Supine	Sitting	Standing
FEV_1_%	33.8 (14.6)	37.6 (14.2)	41.7 (16.3)	33.1 (14.5)	36.8 (13.5)	42.4 (14.8)	32.5 (13.4)	35.5 (12.5)	39.8 (16.0)	31.4 (13.9)	34.7 (14.6)	38.6 (14.5)	36.6 (16.3)	41.1 (16.4)	44.4 (18.7)
SpO_2_, %	93.4 (3.0)	94.6 (2.2)	95.2 (2.1)	94.6 (2.0)	95.3 (1.7)	95.9 (1.4)	92.6 (3.6)	94.2 (2.7)	94.6 (2.7)	93.4 (2.4)	94.7 (1.8)	95.3 (2.2)	92.9 (3.0)	94.4 (2.1)	95.2 (2.1)
NRS	2.1 (2.4)	2.4 (2.4)	2.4 (2.4)	3.3 (2.5)	3.2 (2.5)	3.2 (2.7)	2.5 (2.5)	2.9 (2.7)	3.0 (2.4)	0.6 (1.1)	1.6 (1.3)	1.6 (1.5)	1.1 (1.8)	1.4 (2.0)	1.3 (1.8)

Values show mean (standard deviation)

*FEV*_*1*_*%* percentage of predicted forced expiratory volume in 1 s value, *NRS* numerical rating scale for assessing postoperative pain, *PO6h* after 6 h from the end of surgery, *POD* postoperative day, *PODR6* 6 h after chest drain removal, *SpO*_*2*_ oxygen saturation

Mean FEV_1_% increased from supine to sitting 3.7% (95% CI 2.1 to 5.4, *P* = 0.012) on PO6h, 3.0% (95% CI 1.4 to 4.6, *P* = 0.014) on POD 1, 3.3% (95% CI 0.5 to 6.1, *P* = 0.014) on POD 2 and 4.5% (95% CI 3.0 to 6.1, *P* = 0.013) on PODR6. From supine to standing, there was a further increase to 9.3% (95% CI 6.4 to 12.1, *P* = 0.004) on PO6h, 7.3% (95% CI 4.6 to 10.1, *P* = 0.005) on POD 1, 7.2% (95% CI 2.2 to 12.1, *P* = 0.005) on POD 2 and 7.8% (95% CI 5.1 to 10.5, *P* = 0.004) on PODR6, but without a difference in FEV_1_% from sitting to standing (Fig. 2A).

Mean SpO_2_ from supine to sitting increased 0.7% (95% CI 0.1 to 1.3, *P* = 0.002) on PO6h, 1.6% (95% CI 1.0 to 2.1, *P* = 0.001) on POD 1, 1.3% (95% CI 0.3 to 2.3, *P* = 0.001) on POD 2 and 1.5% (95% CI 1.0 to 2.1, *P* = 0.001) on PODR6. From supine to standing, there was an even more pronounced increase of 1.3% (95% CI 0.4 to 2.2, *P* = 0.002) on PO6h, 2.0% (95% CI 1.3 to 2.9, *P* = 0.001) on POD 1, 1.9% (95% CI 0.4 to 3.4, *P* = 0.001) on POD 2 and 2.3% (95% CI 1.4 to 3.1, *P* = 0.001) on PODR6. Changing posture from sitting to standing did not significantly increase mean SpO_2_ (Fig. 2B).

Postoperative pain did not increase during mobilisation (Fig. 2C).

## Discussion

Summarising, these first data on the effect of well-defined mobilisation (change of posture from supine to sitting to standing) after fast-tracking VATS lobectomy confirms previous findings from non-ERAS open abdominal surgery with improved oxygen saturation during mobilisation (Mynster et al. 1996). Although early mobilisation has been advocated as part of an ERAS programme from the very beginning (Kehlet 1997), detailed data on the degree of mobilisation are scarce (Basse et al. 2002; Fiore Jr. et al. 2017; Balvardi et al. 2021). However, an early mobilisation programme with objective assessment of degree of mobilisation (step count) and aiming at 200 m walk/day by staff-assisted transfers did not find any positive outcomes at 4 weeks postop in ERAS colonic programme (Fiore Jr. et al. 2017). Similarly, from the same trial, this mobilisation regime did neither positively influence pulmonary function and outcome from days 1–3 postoperatively. However, there was no mentioning of a potential association between pulmonary function during the mobilisation. Also, the compliance with the mobilisation programmes was not complete or analysed in detail (Balvardi et al. 2021).

Since bed rest per se may have detrimental effects on several organ systems (Harper and Lyles 1988), early mobilisation continues to be rational to improve function such as muscle function and decreased risk of thromboembolic complications. However, the problem to show the exact differential effect of early postoperative mobilisation on outcome has been difficult and probably not realistic due to the multimodal interventional nature of enhanced recovery programmes (Kehlet 2020). Nevertheless, the present data and the similar observations from non-ERAS open abdominal surgery (Mynster et al. 1996; Basse et al. 2002) serve as a major stimulus for the integration of enforced early mobilisation in perioperative care and which may be of special value when performing pulmonary surgery with an inherited risk of pulmonary complications (atelectasis, pneumonia, respiratory failure, etc.) and need for oxygen support (Kaneda et al. 2007). Consequently, the enforced early postoperative mobilisation should despite some negative long-term data from an ERAS colonic programme (Fiore Jr. et al. 2017; Balvardi et al. 2021) be prioritised in nursing care and studied in more detail with objective monitoring of mobilisation in VATS and other pulmonary procedures. In this context, reasons for not being mobilised should be analysed with regard to organisational vs. patient-related factors. Importantly, early mobilisation may be hindered by early orthostatic intolerance (Jans and Kehlet 2017, Nakada et al. 2021) calling for further studies on the pathogenic mechanisms and prevention (Jans and Kehlet 2017; Kehlet 2020).

The strength of this study includes the detailed methodology with well-defined measurements in different body positions. Despite of a small sample size, there were valid outcomes without missing data. The limitations include a lack of a formal power calculation being a first and explanatory pilot study. Furthermore, the activity may not have had enough discrimination between sitting and standing, and a longer walk procedure may have improved the design. Finally, the clinical outcome implementations of the relatively small changes in FEV_1_% and SpO_2_ during mobilisation need to be addressed in future larger trials.

## Conclusion

In summary, these first detailed data on the effect of mobilisation from supine into sitting and standing position on lung function and oxygenation after fast-tracking VATS lobectomy support the value of early mobilisation and calling for larger outcome studies with a well-defined enhanced mobilisation program.

## Data Availability

Data are available from the first author Lin Huang (lin.huang@regionh.dk) on reasonable request.

## References

[CR1] Balvardi S, Pecorelli N, Castelino T, Niculiseanu P, Alhashemi M, Liberman AS, Charlebois P, Stein B, Carli F, Mayo NE, Feldman LS, Fiore JF (2021). Impact of facilitation of early mobilization on postoperative pulmonary outcomes after colorectal surgery: a randomized controlled trial. Ann Surg.

[CR2] Basse L, Raskov HH, Hjort JD (2002). Accelerated postoperative recovery programme after colonic resection improves physical performance, pulmonary function and body composition. Br J Surg.

[CR3] Batchelor TJP, Rasburn NJ, Abdelnour-Berchtold E, Brunelli A, Cerfolio RJ, Gonzalez M, Ljungqvist O, Petersen RH, Popescu WM, Slinger PD, Naidu B (2019). Guidelines for enhanced recovery after lung surgery: recommendations of the Enhanced Recovery After Surgery (ERAS®) Society and the European Society of Thoracic Surgeons (ESTS). Eur J Cardiothorac Surg.

[CR4] Bonnet F, Bourgain JL, Matamis D, Tesseire B, Viars P (1988). The influence of position on ventilation-perfusion distribution after abdominal surgery. Acta Anaesthesiol Scand.

[CR5] Craig DB (1981). Postoperative recovery of pulmonary function. Anesth Analg.

[CR6] Craig DB, Wahba WM, Don HF, Couture JG, Becklake MR (1971). “Closing volume” and its relationship to gas exchange in seated and supine positions. J Appl Physiol.

[CR7] Fiore JF, Castelino T, Pecorelli N (2017). Ensuring early mobilization within an enhanced recovery program for colorectal surgery: a randomized controlled trial. Ann Surg.

[CR8] Gharaibeh A, Koppikar S, Bonilla-Escobar FJ (2014). Strengthening the Reporting of Observational Studies in Epidemiology (STROBE) in the International Journal of Medical Students. Int J Med Stud.

[CR9] Hansen HJ, Petersen RH (2012). Video-assisted thoracoscopic lobectomy using a standardized three-port anterior approach - the Copenhagen experience. Ann Cardiothorac Surg.

[CR10] Harper CM, Lyles YM (1988). Physiology and complications of bed rest. J Am Geriatr Soc.

[CR11] Harris PA, Taylor R, Thielke R, Payne J, Gonzalez N, Conde JG (2009). Research electronic data capture (REDCap)--a metadata-driven methodology and workflow process for providing translational research informatics support. J Biomed Inform.

[CR12] Hertzog MA (2008). Considerations in determining sample size for pilot studies. Res Nurs Health.

[CR13] Hsu HO, Hickey RF (1976). Effect of posture on functional residual capacity postoperatively. Anesthesiology..

[CR14] Jans O, Kehlet H (2017). Postoperative orthostatic intolerance: a common perioperative problem with few available solutions. Can J Anaesth.

[CR15] Kaneda H, Saito Y, Okamoto M, Maniwa T, Minami KI, Imamura H (2007). Early postoperative mobilization with walking at 4 hours after lobectomy in lung cancer patients. Gen Thorac Cardiovasc Surg.

[CR16] Kehlet H (1997). Multimodal approach to control postoperative pathophysiology and rehabilitation. Br J Anaesth.

[CR17] Kehlet H (2020). Enhanced postoperative recovery: good from afar, but far from good?. Anaesthesia..

[CR18] Meyers JR, Lembeck L, O'Kane H, Baue AE (1975). Changes in functional residual capacity of the lung after operation. Arch Surg.

[CR19] Mynster T, Jensen LM, Jensen FG (1996). The effect of posture on late postoperative oxygenation. Anaesthesia..

[CR20] Nakada T, Shirai S, Oya Y, Takahashi Y, Sakakura N, Ohtsuka T, Kuroda H (2021). Four hours postoperative mobilization is feasible after thoracoscopic anatomical pulmonary resection. World J Surg.

[CR21] Wildgaard K, Petersen RH, Hansen HJ, Møller-Sørensen H, Ringsted TK, Kehlet H (2012). Multimodal analgesic treatment in video-assisted thoracic surgery lobectomy using an intraoperative intercostal catheter. Eur J Cardiothorac Surg.

